# Identifying changes in EEG information transfer during drowsy driving by transfer entropy

**DOI:** 10.3389/fnhum.2015.00570

**Published:** 2015-10-23

**Authors:** Chih-Sheng Huang, Nikhil R. Pal, Chun-Hsiang Chuang, Chin-Teng Lin

**Affiliations:** ^1^Brain Research Center, National Chiao-Tung UniversityHsinchu, Taiwan; ^2^Institute of Electrical Control Engineering, National Chiao-Tung UniversityHsinchu, Taiwan; ^3^Electronics and Communication Sciences Unit, Indian Statistical InstituteCalcutta, India; ^4^Faculty of Engineering and Information Technology, University of Technology SydneySydney, NSW, Australia

**Keywords:** drowsy driving, EEG, effective connectivity, transfer entropy, driving performance

## Abstract

Drowsy driving is a major cause of automobile accidents. Previous studies used neuroimaging based approaches such as analysis of electroencephalogram (EEG) activities to understand the brain dynamics of different cortical regions during drowsy driving. However, the coupling between brain regions responding to this vigilance change is still unclear. To have a comprehensive understanding of neural mechanisms underlying drowsy driving, in this study we use transfer entropy, a model-free measure of effective connectivity based on information theory. We investigate the pattern of information transfer between brain regions when the vigilance level, which is derived from the driving performance, changes from alertness to drowsiness. Results show that the couplings between pairs of frontal, central, and parietal areas increased at the intermediate level of vigilance, which suggests that an enhancement of the cortico-cortical interaction is necessary to maintain the task performance and prevent behavioral lapses. Additionally, the occipital-related connectivity magnitudes monotonically decreases as the vigilance level declines, which further supports the cortical gating of sensory stimuli during drowsiness. Neurophysiological evidence of mutual relationships between brain regions measured by transfer entropy might enhance the understanding of cortico-cortical communication during drowsy driving.

## Introduction

According to the 2009 Sleep in America Poll (Foundation, 2009), 54% of adults experience drowsiness during driving, and 28% of drivers even nod off or fall asleep at the wheel. Driving under the influence of fatigue/drowsiness often results in serious car accidents. Therefore, to prevent accidents, a comprehensive understanding of the neurophysiological markers of declining vigilance in drivers is necessary and it may provide insight into the mechanism underlying the drowsy driving.

Several neuroscience studies using EEG (Baulk et al., 2001; Lal and Craig, 2002; Banks et al., 2004; Campagne et al., 2004; Eoh et al., 2005; Lal and Craig, 2005; Rosario et al., 2010; Chuang et al., 2012) have revealed that changes in alertness during driving are linked to changes in global brain dynamics. For example, in our previous study (Chuang et al., 2012) we have observed synchronized EEG spectral dynamics between spatially non-contiguous areas of the brain when drowsiness occurs. These growing evidences suggest that changes in effective connectivity in a specific cortico-cortical pathway may be a sensitive neurophysiological signature for changes in alertness.

Over the past decade, many studies tried to elucidate the network level mechanisms of neurocognitive function by using effective connectivity measures (Korzeniewska et al., 2003; Blinowska et al., 2004; Astolfi et al., 2007; Supp et al., 2007; Liu et al., 2010; Vicente et al., 2011; Liu et al., 2012). One of the most widely used measures to study effective connectivity in cognitive neuroscience is Granger Causality (GC); (Granger, 1969). GC and its extensions, such as directed transfer function and partial directed coherence (Korzeniewska et al., 2003; Blinowska et al., 2004; Astolfi et al., 2007; Supp et al., 2007; Liu et al., 2010; Vicente et al., 2011; Liu et al., 2012), essentially model stochastic processes using regression (Sabesan et al., 2009). However, there are three prerequisites to ensure a proper use of GC (Vicente et al., 2011). First, the interaction between two signals should be approximately linear. Second, the observations should have relatively low levels of noise. Third, the cross-talk between signals should be low. However, the interactions between brain signals are usually non-linear. Moreover, due to volume conduction the original causally-related brain signal from a single source and artifacts are mixed into several EEG channels, leading to difficulty in estimating information flow between brain regions on a sensor-space. Transfer entropy (TE) is an alternative measure of effective connectivity for neurosciences (Gourévitch and Eggermont, 2007; Sabesan et al., 2009; Besserve et al., 2010; Vakorin et al., 2010; Vicente et al., 2011; Lee et al., 2012). In contrast to GC, TE is a model-free measure based on information theory that does not require a model of the interaction. TE has demonstrated its robustness against volume conduction as well as its effectiveness in revealing non-linear interactions between brain regions (Lindner et al., 2011; Vicente et al., 2011).

In this study, we have used transfer entropy to examine the association between effective connectivity dynamics and drowsiness-associated performance changes during a sustained-attention driving experiment. Each subject participated in a virtual reality based dynamic driving simulator, in which EEG signals and data on subject's behavior were recorded simultaneously. Our study reveals the effective connectivity between different brain regions using changes in behavioral data and associated information transfer as measured from EEG signal.

## Materials and methods

### Participants and EEG acquisition

Twelve healthy male adults aged 20–30 years were recruited to participate in the sustained-attention driving experiment. All subjects were required to have driving license and good driving habits. None of the participants had a history of psychological disorders. All participants were instructed to sustain their attention to perform the lane-keeping task in the afternoon after lunch without breaks. Prior to the experiment, all participants completed a consent form stating their clear understanding of the experimental protocol which had been approved by Institutional Review Broad of Taipei Veterans General Hospital, Taiwan.

A wired EEG cap with 32 Ag/AgCl electrodes, including 30 EEG electrodes and two reference electrodes (opposite lateral mastoids) was used to record the electrical activity of the brain from the scalp during the driving task. The EEG electrodes were placed according to a modified international 10–20 system. The contact impedance between all electrodes and the skin was kept < 5 kΩ. The EEG recordings amplified by Scan SynAmps2 Express system (Compumedics Ltd., VIC, Australia) were digitized at 500 Hz (resolution: 16 bits). Before data analysis, the raw EEG data were preprocessed by the following steps: First, we use a digital band-pass (1–50 Hz) zero-phase FIR filter (the eegfilt.m routine from the EEGLAB toolbox, Delorme and Makeig, 2004) to remove the power line noise and low-frequency drift. Second, the signals are down-sampled to 250 Hz to reduce the volume of data. Finally, we do manual removal of some artifacts such as random and persistent disturbance from body motion, eye movement, eye blinking, muscle activity, EEG channel malfunction, and environmental noise.

### Sustained-attention driving experiment

**Figure 1:**
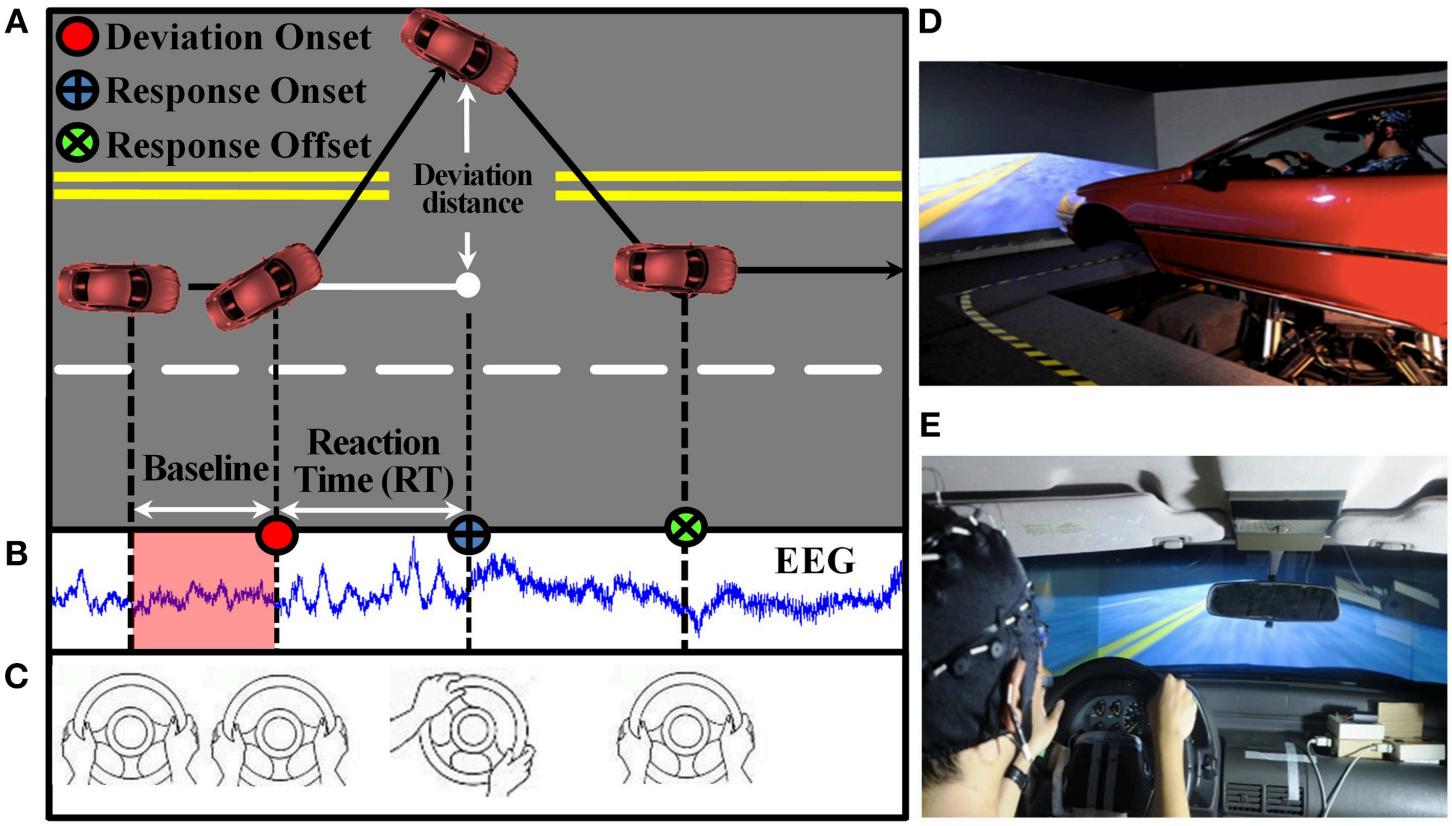
**Sustained-attention driving task**. **(A)** Event-related lane-deviation paradigm. **(B–C)** EEG and behavior were recorded simultaneously. **(D)** The experimental paradigm was implemented in a VR-based driving simulator, where a 3-D surrounding view simulated a monotonous highway scene and a vehicle was mounted on a six degree-of-freedom dynamic platform. **(E)** The car speed was fixed at 100 km/h and the subject was asked to keep the car cruising on the central of the lane.

This study adopted an event-related lane-departure paradigm (Figure 1A) (Huang et al., 2009) in a virtual-reality (VR) dynamic driving simulator (Figures 1D,E) to quantitatively measure brain EEG dynamics along with the fluctuation of task performance throughout the experiment. All subjects participated in the sustained-attention driving experiment for 1.5 h in the afternoon (13:00–14:00) after lunch, and all of them were asked to keep their attention focused on driving during the entire period. There was no break or resting session. At the beginning of the experiment, a 5 min pre-test was performed to ensure that every subject understood the instructions and they did not suffer from simulator-induced nausea. During this 90 min sustained-attention driving task, the experimental paradigm simulated a nighttime driving on a four-lane highway and lane-departure events were randomly induced to make the car drift away from the original cruising lane toward the left or right sides. Each participant was instructed to quickly compensate for this perturbation by steering the wheel (Figure 1C). To avoid impacts of other factors during the task, participants only reacted to the lane-perturbation event by turning the steering wheel, and they did not have to control the accelerator and brakes pedals in this experiment.

A complete trial in this study, including 1 s baseline, deviation onset, response onset, and response offset, is shown in Figure 1A. EEG signals were recorded simultaneously (Figure 1B). The next trial occurs within an interval of 5–10 s after finishing the current trial in which the subject has to drive back to the center line of the third car lane. If a subject fell asleep during the experiment, there was no feedback to alert him up.

### Connectivity estimation by transfer entropy

Transfer entropy (TE), an information-theoretic measure proposed by Schreiber (2000), is derived from the mutual information theory to assess conditional transition probabilities between two paired processes evolving in time. Consider two simultaneously measured time series generated by random processes *X* and *Y*. We assume that each of these processes can be approximated by a stationary Markov Process of finite order d. Thus, we can reconstruct the state space of the process *X* by a delay embedded vector of dimension *d* with past values. Representing the two time series as *X* = *x*_*t*_ and *Y* = *y*_*t*_, the delay embedded vector is defined as ${\text{x}}_{t}^{d}=\left(x_{t},x_{t-\tau },x_{t-2\tau },\cdots \;,x_{t-\left(d-1\right)\tau }\right)\;$; similar representation can also be made for $y_{t}^{d}$. The dimension of the embedding space is *d*, and the delay is τ. Under the assumption that the system *X* can be approximated by a stationary Markov process of order *d*, the transition probabilities that describe the system are given by:

(1) $$\begin{array}{l} p\left(x_{t+1}|{\text{x}}_{t}^{d}\right). \end{array}$$

The *entropy rate* of the system *X* is the average number of bits that is required to represent an additional state provided all previous states are known. Thus, the entropy rate can be computed as follows

(2) $$\begin{array}{l} h\left(x_{t+u}|{\text{x}}_{t}^{d}\right)=-\underset{{\text{x}}_{t+u},{\text{x}}_{t}^{d}}{\sum }p\left(x_{t+u},{\text{x}}_{t}^{d}\right)\log p\left(x_{t+u}|{\text{x}}_{t}^{d}\right), \end{array}$$

where $p\left(x_{t+u}|{\text{x}}_{t}^{d}\right)=p\left(x_{t+u},{\text{x}}_{t}^{d}\right)∕p\left({\text{x}}_{t}^{d}\right)$,

*u* is the prediction time, and *p*(^*^) is the probability.

If the two processes are independent, there will be no transfer of information and $p\left(x_{t+u}|{\text{x}}_{t}^{d}\right)=p\left(x_{t+u}|{\text{x}}_{t}^{d},{\text{y}}_{t}^{m}\right)$. As proposed by Schreiber (2000), a measure of deviation from this generalized Markov property can be computed using Kullback divergence or mutual information and that is a directed measure of information transfer from *Y* to *X*. The amount of information transferred (i.e., transfer entropy) from process Y to process X, denoted as TE(*Y* → *X*), is computed as (Schreiber, 2000; Hlaváèková-Schindler et al., 2007; Vicente et al., 2011)

(3) $$\begin{array}{l} \text{TE}\left(Y\to X\right)=\sum p\left(x_{t+u},{\text{x}}_{t}^{d},{\text{y}}_{t}^{m}\right)\log\frac{p\left(x_{t+u}|{\text{x}}_{t}^{d},{\text{y}}_{t}^{m}\right)}{p\left(x_{t+u}|{\text{x}}_{t}^{d}\right)}, \end{array}$$

where $y_{t}^{m}=\left\{y_{t},y_{t-\tau },\cdots \;,y_{t-\left(m-1\right)\tau }\right\}$ indicating that the process *X* depends on *m* states of *Y*. The Equation (3) can be rewritten in terms of differential entropy as the following

(4) $$\begin{array}{l} \text{TE}(Y\to X)=H(x_{t}^{d},y_{t}^{m})-H(x_{t+u},x_{t}^{d},y_{t}^{m})\\ \;+H(x_{t+u},x_{t}^{d})-H(X_{t}^{d}). \end{array}$$

Note that TE is inherently asymmetric, that is TE(*Y* → *X*) ≠ TE(*X* → *Y*). In addition, when the processes are mutually independent then TE(*Y* → *X*) = TE(*X* → *Y*) = 0.

The estimation of parameters and the calculation of TE were performed by TRENTOOL (version 2.0.4) (Lindner et al., 2011). Specifically, the TE values were estimated by the *k*-nearest neighbor approach (Kraskov et al., 2004; Vicente et al., 2011), with *k* = 4 as suggested by Kraskov et al. (2004). The embedding delay (τ) was determined based on the Cao criterion (Cao, 1997), and the dimension (*d* and *m*) was obtained by an effective search algorithm (Cormen et al., 2001; Lindner et al., 2011; Vicente et al., 2011). Theiler correction tries to remove autocorrelation effect from the density estimation. For the nearest neighbor search, it discards all samples which are very close in time with respect to a reference point. Here the Theiler correction window (*T*) was set to 1. In this study the prediction time *u* was set to 5. This was determined by finding the maximum value of TE over a set of choices: (5, 10, 20, 40, 60, 80, and 100 ms).

### Behavioral performance

During a 1.5 h-long driving task, participants might suffer from drowsiness from time to time, which leads to performance fluctuations. To objectively and continuously measure participants' driving performance, the reaction time (RT), the elapsed time between onsets of deviation and response (Figure 1A), was calculated. A short RT indicates a good performance on the driving task and vice versa. In this study, subjects were instructed to respond to the stimulus as fast as they could. Therefore, among a group of participants performing the same task, individual characteristics might lead to differences in distribution of RT. For instance, the mean RT for some subjects was found to be lower than 2 s, but for others it was larger than 2 s.

To overcome the individual difference in behavioral performance, RTs of each subject were first normalized dividing by the average of the first 10% of the RTs arranged in ascending order to obtain normalized RTs (Equation 5). This procedure guaranteed that all RTs corresponding to the optimal driving performance (fast RT) collected from different participants would approximately be one.

(5) $$\begin{array}{l} {\text{Normalized}\;\text{RT}}_{i}=\frac{{\text{RT}}_{i}}{<{\text{RT}}_{j}^{\text{top}\;10\text{\%}}>}, \end{array}$$

where *i* = 1, …, *N* represents the trial index, *N* is the number of trials for each subject, and ${\text{RT}}_{j}^{\text{top}\;10\text{\%}}$ represents the *j*th RT in the top 10% of the shortest RTs. The bracket, i.e., < ·>, represents the average value.

**Figure 2:**
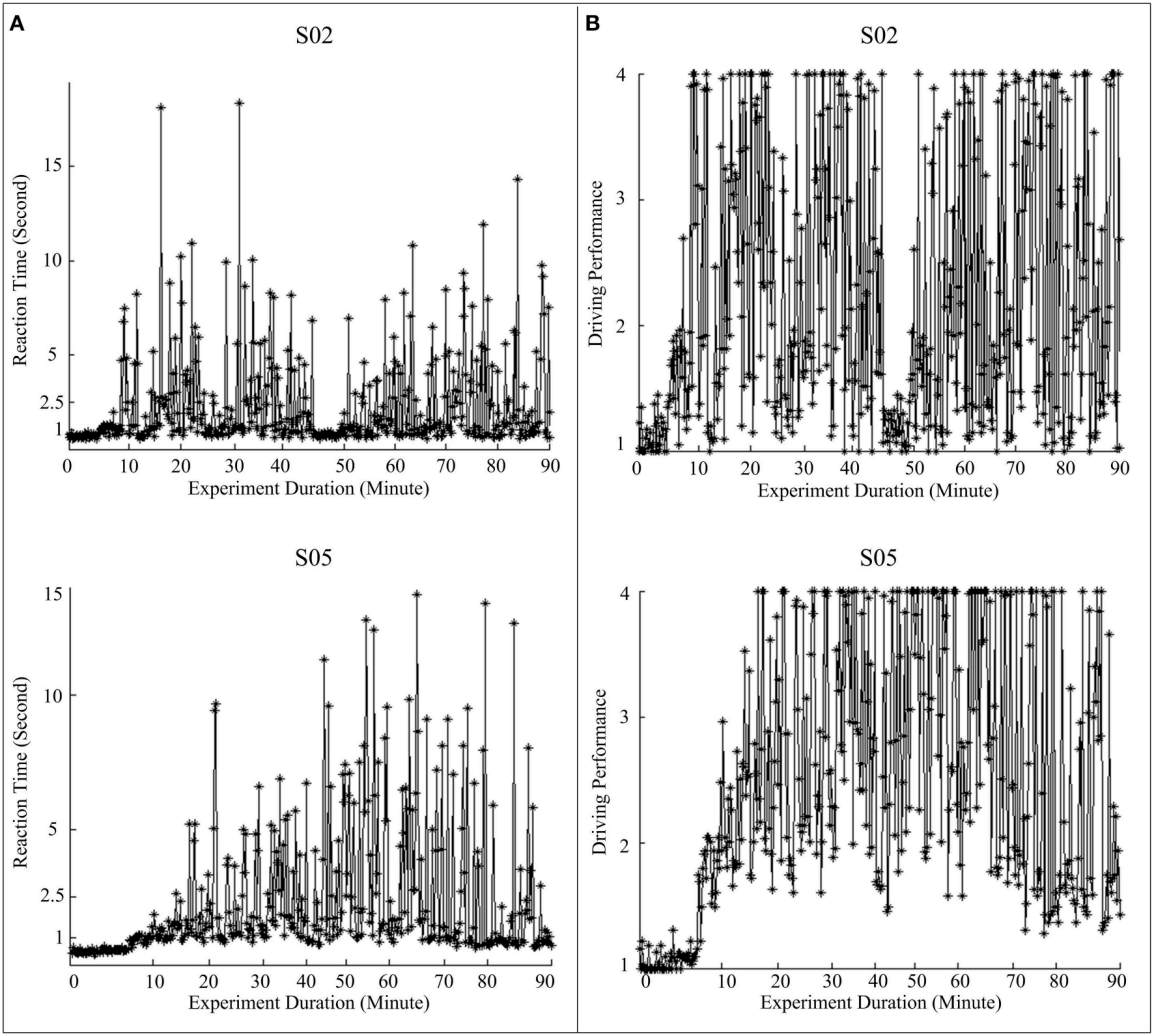
**Fluctuations in behavioral performance during a 1.5 h experiment for two subjects (Top panel: S02, and bottom panel: S05)**. **(A)** Recorded RTs; **(B)** driving performance (DP) converted from RTs using equations (5) and (6).

In our experiment design, the simulated vehicle hits roadside curbs, if the subject does not respond to a perturbation event within 1.5 s for the left side (or within 2.5 s for the right side). If the simulated vehicle hits the roadside curbs, the program does not give any feedback to the driver. Since human physiological system and the experimental paradigm impose no upper bound to the RT, the actual RT varies widely, especially for drowsy subjects. Empirically, if the subjects are under a high vigilance level, the RTs are usually less than 1 s. Keeping this in mind, if a normalized RT is less than 1, we set it to 1 as the person is definitely in an alert state. If the RT is larger than 2.5 s, then the subject is expected to be in a low vigilance level. In fact, in order to study the transition of vigilance level from alert to drowsy, analysis of EEG associated with RT of up to 3 s is very important. However, we have already mentioned that the actual RT can take a very high value, even 200 s. Empirically it is also found that if the RT is more than 4 s, the subject is certainly in a state of very low vigilance. Therefore, to define an index to assess the driving performance (DP), we need to transform the RT in such a manner that RT greater than 4 does not affect much the DP, but the DP is adequately sensitive to RT when RT varies between 1 and 4. The Equation (6) involving a logistic transformation indeed achieves the same. For RT = 1, DP is also 1; when RT = 4, DP is 3.11 and for RT from 3 to infinity, DP varies between 3.11 and 4.08. It is also interesting to observe that up to RT = 2.5 s, DP follows almost a linear relation (DP = 2.26 for RT = 2.5 s), which is indeed desirable. Figure 2 presents the behavioral performances, including actual reaction times and corresponding derived driving performance, during a 1.5 h experiment for two subjects.

(6) $$\begin{array}{l} {\text{DP}}_{i}=-\left(\frac{1+e^{-0.5}}{1-e^{-0.5}}\right)+\left(\frac{2+2e^{-0.5}}{1-e^{-0.5}}\right)\\ \;\left(\frac{1}{1+e^{-0.5\times \text{Normalized }{\text{RT}}_{i}}}\right) \end{array}$$

### EEG signals of interest

Researchers, Huang et al. (2009) and Chuang et al. (2014a) have reported that the independent EEG components corresponding to frontal, central, right motor, left motor, parietal, and occipital region are highly correlated with fatigue, drowsiness, and behavioral lapse. Chuang et al. (2014b) further proposed a brain–computer interface using these independent components to effectively predict the change in the cognitive state. The aforementioned brain regions provide important information concerning drowsy driving. We aim to investigate the transfer of information among different brain regions using EEG signals during drowsy driving. Since volume conduction and superposition of brain source signals to EEG electrodes is linear and there is not much delay involved, independent component analysis (ICA) can be used to find component signals mixed by volume conduction (Whitmer et al., 2010). Therefore, discarding the ICs representing artifacts, the effect of volume conduction can be reduced to some extent. Since in this study, we have used ICA to remove artifacts (non-physiological), it has reduced the effect of volume conduction also. Further, we have applied the time-shift test (Lindner et al., 2011) to identify instantaneous mixing between artifact-free EEG signals from pairs of channels. In fact, after performing the time-shift test, which was implemented by TRENTOOL (version 2.0.4), no instantaneous mixing problem was found in all artifact-free EEG pairs. It is worth noting, however, that the same time-shift test when applied on the raw EEG data (before artifact removal) rejected some pairs of EEG channels. In order to reduce the number of channels and the computational complexity, in this work we chose only six EEG channels, that are the closest to the independent components mentioned above, i.e., Fz (frontal region), Cz (center region), C3 (left motor region), C4 (right motor region), Pz (parietal region), and Oz (occipital region).

### EEG analysis

Our work intends to find the EEG pattern of tonic physiological changes that are associated with changes in vigilance. The EEG activities in the baseline period sufficiently represent the cognitive status in the current trial. For instance, subjects can react to the event quicker under higher vigilance level and vice versa. Therefore, we only analyze physiological EEG changes (the spectral activity and the effective connectivity) in the 1 s baseline period for each trial (tonic analysis); as shown in the red covered part of Figure 1B, and the corresponding DP can be used as an objective index to represent the vigilance level of the current trial. The power spectral activities of EEG signals were calculated by fast Fourier transformation (FFT) for each EEG channel, and the effective connectivity between every pair of the selected EEG channels was estimated by the TE and GC. In this study, the order of GC was determined by using Bayesian Information Criterion (BIC).

### Statistical analysis

The TE values of each subject were first normalized by subtracting the baseline TE value to reduce the individual difference. Here the baseline TE value was obtained by averaging TE values over the trials for which the corresponding DPs were within the best tenth percentile of DP (top 10% DP). Therefore, the normalized TE values are relative-TE values, which represent changes in TE values compared to the baseline TE value. For further analysis, TE values from all subjects were grouped together and sorted by DPs from the best to the worst. The performance-related TE dynamic connectivity was obtained by applying a moving-average filter (window size: 0.5 unit of DP and step size: 0.1 unit of DP) on the DP-sorted TE value. Then, statistical significance was tested by comparing TE values from each window with the first window (DP = 1–1.5) by using Wilcoxon rank-sum test. Results of GC and power spectral activity were also computed in the same manner. Additionally, linear dependence between EEG spectral activities and DPs was measured by Pearson's correlation coefficient (see **Table 2**).

Furthermore, for group analysis, for each subject, three performance groups were defined: optimal (DPs < 2), sub-optimal (2 ≤ DPs ≤ 3), and poor (DPs > 3). We have investigated the difference of TE among performance groups (optimal, sub-optimal, and poor) by One-way repeated ANOVA. These results are summarized in **Table 3**. The Wilcoxon signed-rank test was further used to test the pair wise difference between different performance groups. In order to account for multiple comparisons we have computed the false discovery rate (FDR, Genovese et al., 2002)-adjusted *p*-values.

## Experimental results

### Behavioral performance

**Figure 3:**
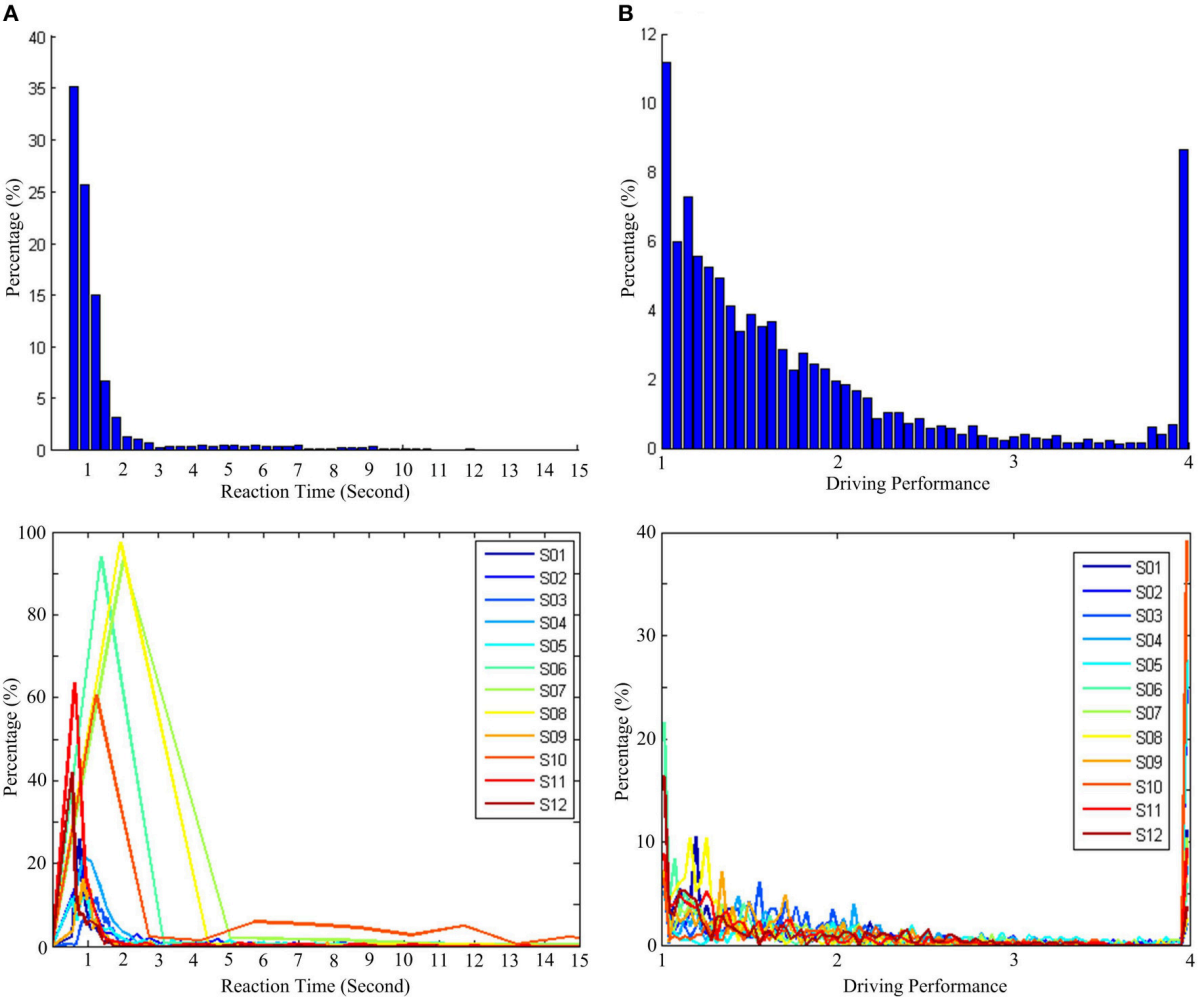
**Distribution of reaction time (RT) and driving performance (DP). (A)** Histogram of RT. **(B)** Histogram of DP. Top panel presents the distribution for all subjects, and bottom panel presents the distributions for each subject.

Table 1 presents the descriptive statistics, including the arithmetic mean, the standard deviation, second quartile, and the maximum value of RTs and DPs. Figures 3A,B show the distributions of the RT recorded from all sessions and the corresponding values of the DP derived by Equations (5) and (6), respectively. For visual simplicity, we display herein just 15 s on the x-axis (RT)—the actual maximum is about 300 s (see the maximum actual RT of S07 in Table 1). Since subjects were instructed to minimize the reaction time in response to vehicle perturbations, one could predict that the distribution of the RT as well as the DP would be positively skewed. The averages of all RTs and DPs from 12 subjects were 2.38 ± 6.91 (SD) and 1.83 ± 0.81 (SD), respectively. The standard deviations are indicative of the variability of the RTs and DPs. Additionally, due to the constraint on the RT-DP conversion, the distribution of the DP is found to be less skewed than that of the RT. Thus, the transformation of RT to DP is a useful trick to deal with the individual difference in driving performance, and the DP can be used as an objective behavioral measurement to characterize vigilance level.

**Table 1:** **Task performance for all subjects**.

**SC**	**S01**	**S02**	**S03**	**S04**	**S05**	**S06**	**S07**	**S08**	**S09**	**S10**	**S11**	**S12**
NOTs	314	465	430	278	424	251	202	232	267	222	526	478
**ACTUAL RT (UNIT: SECOND)**												
Mean	1.60	2.23	1.38	1.81	2.33	2.84	3.27	2.50	1.41	6.78	1.42	0.98
SD.	2.00	2.46	0.68	2.50	2.53	14.56	21.62	16.72	1.45	14.53	2.70	1.13
Q2	0.85	1.17	1.29	1.15	1.22	0.62	0.87	0.95	0.99	1.29	0.69	0.67
Max	11.30	18.32	8.39	22.52	14.04	178.07	304.24	250.98	10.05	150.13	29.62	11.02
**DERIVED DP**												
Mean	1.71	2.19	1.61	1.84	2.41	1.49	1.83	1.37	1.70	2.57	1.66	1.57
SD.	0.88	1.06	0.45	0.75	1.02	0.75	0.83	0.52	0.73	1.17	0.82	0.67
Q2	1.32	1.79	1.56	1.63	2.14	1.22	1.55	1.21	1.47	2.11	1.34	1.32
Max	4.00	4.00	4.00	4.00	4.00	4.00	4.00	4.00	4.00	4.00	4.00	4.00

The mean value, the standard deviation, the second quartile, and the maximum value of actual RT and derived DP for all 12 subjects.

SC represents the code of subject. NOTs represent number of trials.

Mean represents the arithmetic mean value. SD represents the standard deviation.

Q2 represents second quartile (median). Max represents the maximum value.

### DP-sorted EEG spectral perturbations

**Figure 4:**
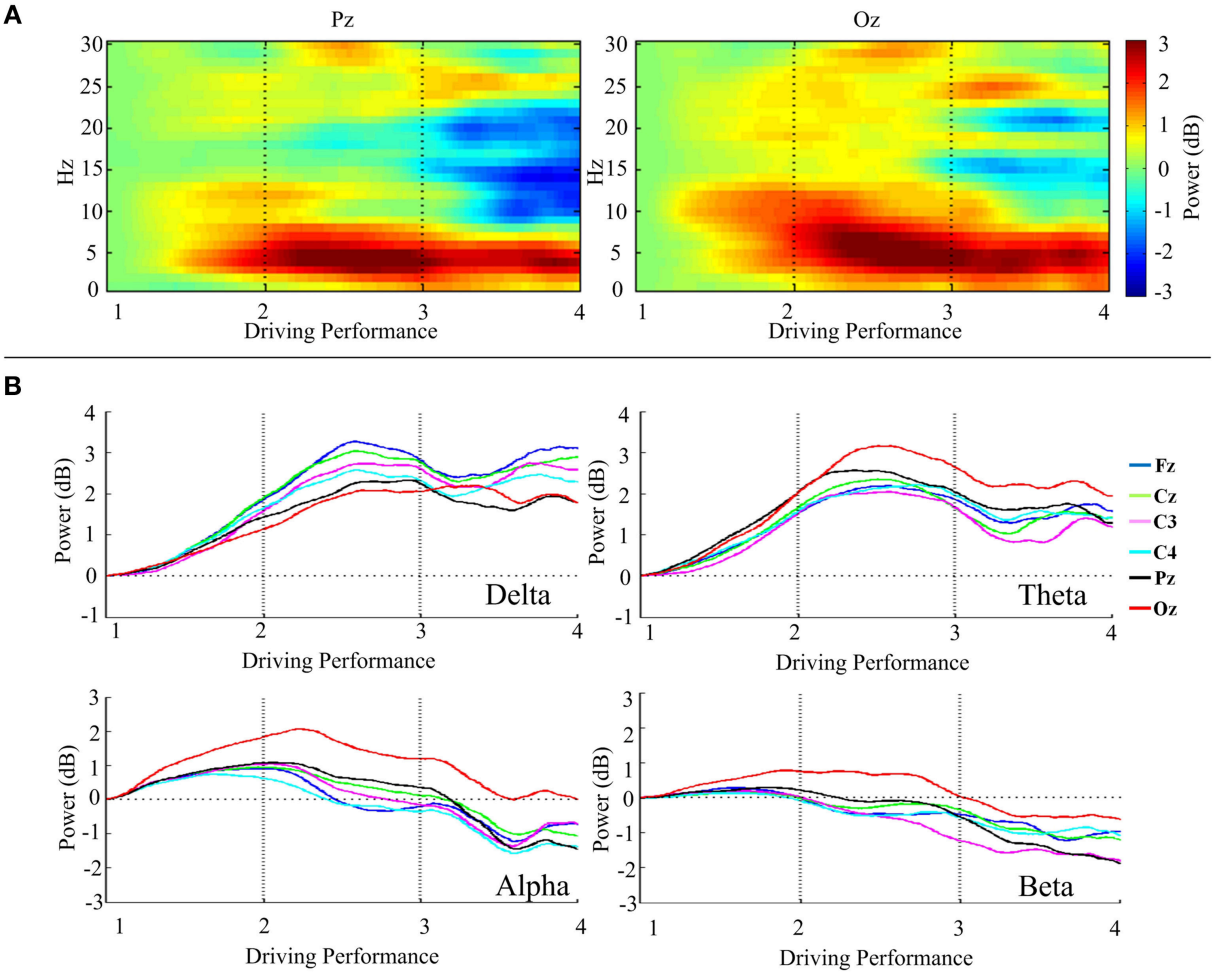
**Relationship between the DP and the EEG power spectra**. **(A)** DP-sorted EEG spectra of Pz and Oz. **(B)** Trends of the DP-sorted delta, theta, alpha, and beta band spectral power of Fz, Cz, C3, C4, Pz, and Oz. The power spectral estimates of all trials are horizontally staked according to the DP from high to low (i.e., from DP = 1 to DP = 4).

We would like to justify the relationship between EEG spectral activities and DPs. For this, in Table 2 we present the Pearson's correlation coefficient between four band powers (delta (1–4 Hz), theta (5–7 Hz), alpha (8–12 Hz), and beta (13–20 Hz) band) and DPs. Figure 4A presents the DP-sorted EEG spectra of Pz and Oz, which are estimated by FFT. Figure 4A reveals that the fluctuations in DP are correlated with concurrent perturbations in EEG power spectra. Figure 4B depicts the trends of the DP-sorted delta (1–4 Hz), theta (5–7 Hz), alpha (8–12 Hz), and beta (13–20 Hz) band spectral power. DP-sorted delta and theta spectral activities exhibit a strong positive correlation, and DP-sorted beta spectral activities exhibit a strong negative correlation, with changes in DPs for all EEG channels considered.

**Table 2:** **Pearson correlation coefficient between the band power and the DP**.

	**Fz**	**Cz**	**C3**	**C4**	**Pz**	**Oz**
Delta	0.8475	0.8329	0.8629	0.8436	0.7783	0.8852
Theta	0.6465	0.5091	0.4479	0.6232	0.4995	0.6705
Alpha	−0.8261	−0.7792	−0.7907	−0.8869	−0.7373	−0.3510
Beta	−0.7056	−0.7048	−0.9427	−0.9465	−0.7795	−0.4771

### DP-sorted EEG connectivity changes

**Figure 5:**
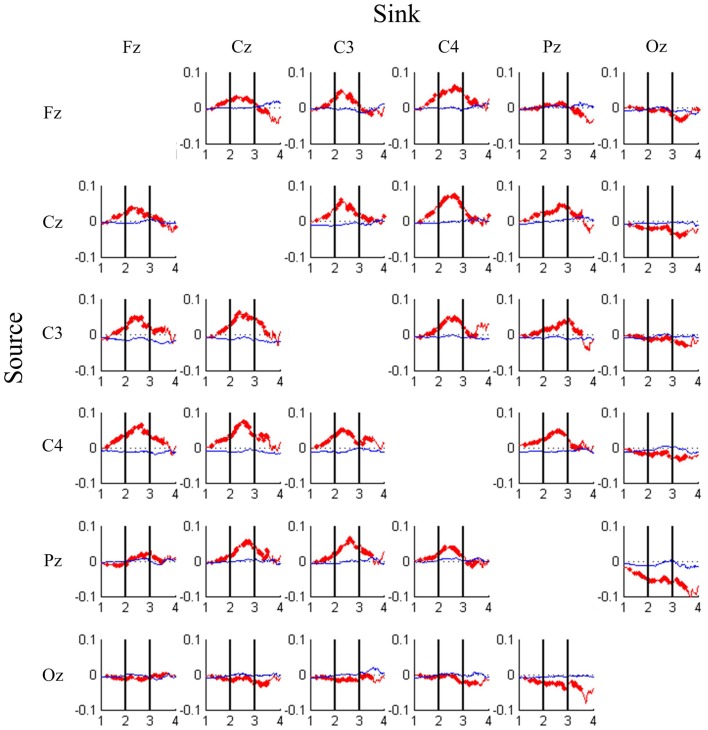
**DP-sorted connectivity magnitudes between pairs of EEG channels estimated by transfer entropy (red trace) and Granger causality (blue trace) analyses, where causality is from rows to columns**. Each cell plots changes in connectivity magnitude (y-axis) from high (DP = 1) to low (DP = 4) task performance (x-axis). Bold line indicates that the FDR-adjusted *p*-value is less than 0.001.

Figure 5 shows the dynamics of the strength of connectivity [i.e., TE values (red color) and GC values (blue color)] between all pairs of EEG channels across different levels of driving performance. In Figure 5, compared with the baseline TE, significant DP-related TE perturbations are highlighted with bold line (Wilcoxon rank-sum test, FDR-adjusted *p* < 0.001). An inverted-U shaped change in TE values is observed in Fz-, Cz-, C3-, C4- and Pz-associated connectivity. These connectivity strengths peak at DP = 2.5 and begins to peak-off with decline in performance. Another interesting observation is that the information transfer monotonously decreases with the decline in DP for most of the Oz-related connectivity pairs, especially in Pz-Oz, and Oz-Pz. It is worth noting here that GC did not exhibit much change when DP varied from the best to the worst.

### Differences in connectivity between distinct categories of driving performance

**Figure 6:**
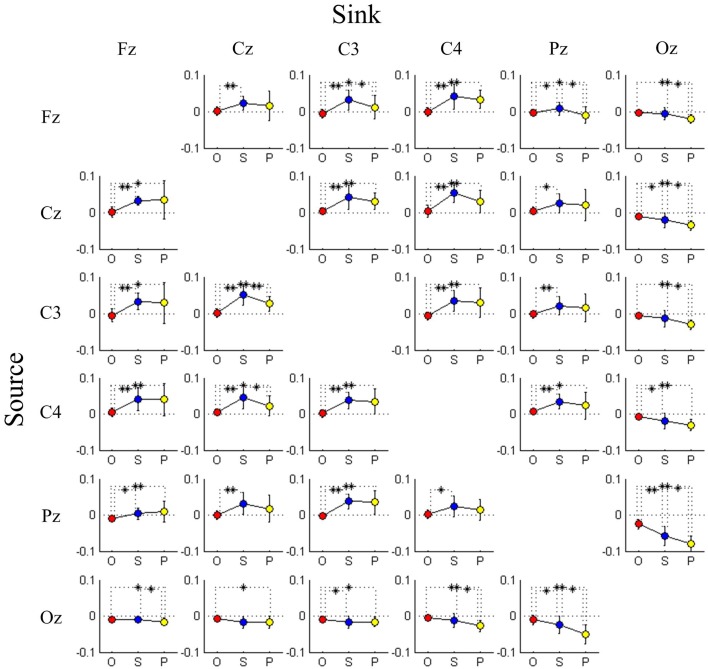
**Comparison of connectivity magnitude between three performance groups estimated by transfer entropy, where causality is from rows to columns**. Each cell plots changes in connectivity magnitude from optimal group (O; red marker) through sub-optimal group (S; blue marker) to poor group (P; yellow marker). Error bars indicate standard deviations. ^*^FDR-adjusted *p* < 0.05 and ^**^FDR-adjusted *p* < 0.01.

As mentioned in the previous section, we have observed two interesting phenomena from the DP-sorted EEG connectivity in Figure 5. Further, in this study we provide a group analysis considering the three performance groups: optimal, sub-optimal, and poor. Table 3 presents the *F*-ratio and *p*-value from One-way repeated measures ANOVA considering the three driving performance groups. Table 3 reveals that for 17 of the 25 pairs of channels, the *p*-value is less than 0.05. The Wilcoxon signed-rank test is further used to test the pair wise difference between performance groups. For dealing with the problems of multiple comparisons, we have used corrections for false discovery rate (FDR) to obtain the FDR-adjusted *p*-value. Figure 6 depicts the significant difference (Wilcoxon signed-rank test) in connectivity magnitude between any two performance groups. Figure 6 also provides strong evidences in support of the inverted-U shaped relation from high to low DP in most of the discovered connections (except the Oz-related pairs). The strength of connectivity in Oz-related pairs declines monotonously from high to low DP.

**Table 3:** **Results of One-way repeated measures ANOVA, and the factor is groups defined by driving performance**.

		**Sink**					
		**Fz**	**Cz**	**C3**	**C4**	**Pz**	**Oz**
Source	Fz		2.1445 (0.1332)	6.4832 (0.0042)	8.9318 (0.0008)	3.0847 (0.0591)	6.1103 (0.0055)
	Cz	4.1317 (0.0250)		8.3834 (0.0011)	12.3923 (0.0001)	1.5133 (0.2351)	8.4316 (0.0011)
	C3	4.0315 (0.0271)	15.9315 (0.0000)		7.5128 (0.0020)	2.2705 (0.1192)	7.0710 (0.0028)
	C4	5.0909 (0.0118)	7.5706 (0.0020)	7.4827 (0.0021)		3.8722 (0.0309)	6.6885 (0.0036)
	Pz	2.9248 (0.0677)	3.9157 (0.0298)	12.0976 (0.0001)	2.5512 (0.0933)		19.8243 (0.0000)
	Oz	2.8563 (0.0718)	1.8480 (0.1735)	1.0822 (0.3506)	7.4341 (0.0022)	9.8100 (0.0005)	

The value in each cell is the F-ratio with degrees of freedom (2, 33), and the number inside the bracket is the p-value. Gray background color indicates that the p-value is less than 0.05.

## Discussion

### Behavioral indicator of drowsiness

In this study, a 90 min driving experiment was designed on a stimulated monotonous driving task to realize drowsy driving. Our data were collected in the early afternoon (13:00–14:00) after subjects finished their lunch when the circadian rhythm related to sleepiness was at its peak (Ferrara and De Gennaro, 2001). Most drivers experience fatigue and exhibit low vigilance during a long and monotonous driving at nighttime (Campagne et al., 2004) or after lunch without any rest (Takahashi and Arito, 2000). When people are fatigue, they usually experience difficulty in maintaining the performance on the task at an adequate level (Boksem et al., 2005; Liu et al., 2010), and consequently fail in engaging in the task (Huang et al., 2009; Moeller et al., 2012). The reaction time is also found to be positively related with the Karolinska sleepiness scale (KSS), which is commonly used for assessment of sleepiness and fatigue (Baulk et al., 2001). In this study, the video surveillance and the driving reaction time also indicated that it was difficult for the participants to sustain their attention on the driving task in this simulated driving environment. Additionally, when vigilance was high (a very low RT), subjects were hardly unaware of lane-perturbation events accompanying visual and kinesthetic stimulus generated from the immersive virtual-reality experiment. On the other hand, severe decline in vigilance as well as falling asleep leading to roadside collisions were found to occur when the RT was high (slow response).

To investigate the relation between fluctuations in driving performance and the concurrent changes in the EEG spectrum, our study revealed evidences in support of close and specific links between cortical brain activities (via changes in EEG spectral power) and driving performance. The correlations of DP with delta and theta spectral power are particularly found to be strong for the posterior and occipital channels, which are consistent with findings in similar recent studies (Lal and Craig, 2002; Huang et al., 2009; Lin et al., 2012; Chuang et al., 2014b; Wascher et al., 2014). Therefore, these evidences demonstrate that the DP is correlated with EEG patterns of drowsiness and DP is a reasonable indicator for representing vigilance levels.

### Task performance-related changes of connectivity

During the transition from optimal to poor task performance, subjects suffered from declining vigilance and fatigue and struggled to avoid behavioral lapses. Under such circumstances, more efforts were needed by subjects to keep themselves engaged in the task, as evidenced by the inverted-U shape of the connectivity magnitude across most connections of the anterior brain regions. The similar brain response was also found after sleep deprivation (Szelenberger et al., 2005; Czisch et al., 2012). Additional compensatory resources (Portas et al., 1998; Drummond et al., 2000, 2001, 2004; Drummond and Brown, 2001; Szelenberger et al., 2005) were required for the enhancement of couplings between distinct brain regions. One of the functions of the parietal cortex is to integrate sensory information with motor signals from the motor cortex to accomplish sensorimotor transformations for motor planning and sensory guidance of movements (Fogassi and Luppino, 2005). Therefore, these inverted U-shaped changes in connectivity magnitudes observed around Cz, C3, C4, and Pz sites might imply that the neurophysiological activity of the sensorimotor areas enhanced the brain connectivity to prepare for upcoming traffic events when subjects were under a transition state from high to low vigilance level. In the poor performance group, inspection of the video revealed that subjects were in a drowsy state and even closed their eyes, and often did not respond to perturbations. The results of the DP-sorted connectivity magnitude observed in the occipital area showed a monotonic descending trend of the connectivity, which might be related to the fading of consciousness (Massimini et al., 2005). These reductions of cortico-cortical connectivity might produce a cortical gate that disconnects the brain from the external environment and blocks sensory inputs (Esser et al., 2009).

### Comparison of causality measures

Most EEG studies estimated the independent EEG activations, which are associated with brain source activations, by ICA (Huang et al., 2009; Vakorin et al., 2010; Chuang et al., 2012; Lin et al., 2012; Liu et al., 2012). However, ICA assumes that the subcomponents are statistically independent of each other, and therefore, finding a probabilistic dependence between brain regions using statistically independent signals seems to contradict the hypothesis *per se*! It is questionable that TE, a measure of mutual dependence between two processes assessed by conditional joint probabilities, is an appropriate tool to measure information transfer between independent components. One could expect that TE would find no transfer of information between independent components. To validate this hypothesis, TE between independent EEG components was computed. As expected, the permutation tests showed that the estimated TE between independent EEG components was not significantly different from zero. Regarding causality measurement, most studies have applied Granger causality analysis (Blinowska et al., 2004; Supp et al., 2007; Liu et al., 2010, 2012) in the field of neuroscience to understand the interactions of activated brain regions. We have also applied GC in estimating the changes of performance-related connectivity (see Figure 5). The volume conduction limits the interpretability of sensor-space connectivity while using Granger causality analysis (Haufe et al., 2013). On the contrary, consistent with the findings in a previous study (Vicente et al., 2011), TE showed its robustness against false positives caused by volume conduction. Compared with GC measurement, the performance-related dynamic connectivity could be well-described by TE.

### Limitations

Despite its usefulness, the use of TE in this study has certain limitations that may be a major obstacle in interpreting the results in terms of physiological changes. The EEG activity of the brain is generally non-stationary and is accompanied by noise that is neither Gaussian nor white (Friman et al., 2001; von Bünau et al., 2009). The measured EEG signals are non-stationary because of the inherent non-stationary dynamics of the brain (von Bünau et al., 2009). Therefore, applying advanced techniques, such as ensemble method (Wollstadt et al., 2014), might be more effective in analyzing such non-stationary data. Although TE is reasonably robust to volume conduction it is not completely immune to it. Faes et al. (2013) considered instantaneous effects as non-physiological because primarily they are caused by artifacts of volume conduction and suggested corrections for volume conduction. In this study, we have used ICA to remove such artifacts (non-physiological) and that has mitigated the effect of volume conduction.

## Conclusion

TE analysis was applied to EEG signals to detect changes in the effective connectivity and to correlate these with changes in the driving performance in a sustained-attention driving experiment. This study has demonstrated that EEG pattern of cortico-cortical connectivity correlated with behavioral lapses is a reasonable indicator for representing vigilance levels. The results obtained using TE have revealed that an inverted U-shaped change in the strength of connectivity dominated during the transition from alert to drowsy state. This result provides evidences in support of a recruitment mechanism to establish couplings between broad regions of the brain to maintain behavioral performance against declining vigilance. Additionally, a monotonically decreasing magnitude of the Oz-Pz connectivity was observed suggesting suppression of propagation of brain activity resulting in a disconnection between internal awareness and the external environment. Neurophysiological evidences of changes in connectivity provided further insight into the distributed brain dynamics, which ultimately shed light on the characteristics of brain-behavior relations in an operational environment.

## Funding

This work was supported in part by the UST-UCSD International Center of Excellence in Advanced Bio-engineering sponsored by the Taiwan National Science Council I-RiCE Program under Grant Number: MOST 103-2911-I-009-101, in part by MOST 104-2627-E-009-001, in part by the Aiming for the Top University Plan, under Contract: 104W963, and in part by the Army Research Laboratory: W911NF-10-2-0022. The views and the conclusions contained in this document are those of the authors and should not be interpreted as representing the official policies, either expressed or implied, of the Army Research Laboratory or the U.S Government. The U.S Government is authorized to reproduce and distribute reprints for Government purposes notwithstanding any copyright notation herein.

### Conflict of interest statement

The authors declare that the research was conducted in the absence of any commercial or financial relationships that could be construed as a potential conflict of interest.
